# Innovative technological resources for Alzheimer's disease care management: A scoping review

**DOI:** 10.1177/20552076251407129

**Published:** 2026-02-20

**Authors:** Maria Almeida, Marta Campos Ferreira, Carla Silva Fernandes

**Affiliations:** 1 112048 Faculty of Engineering of the University of Porto, Portugal; 2 112123 INESC TEC – Institute of Systems and Computer Engineering, Portugal; 3 School of Health, Polytechnic Institute of Viana do Castelo (ESS-IPVC), Portugal; 4 Research Center for Health Technologies and Services (RISE@health), Porto, Portugal; 5 Association ADIT Games, Portugal

**Keywords:** Dementia, Alzheimer's, health app, mobile health, management

## Abstract

**Objective:**

The aim of this scoping review was to map and describe the technological tools reported in the literature that have been designed for care management in Alzheimer's disease, with a particular focus on supporting patients living with the condition, their families, caregivers, and healthcare professionals.

**Methods:**

The review was conducted in accordance with the PRISMA (Preferred Reporting Items for Systematic Reviews and Meta-Analyses) framework. A comprehensive literature search was performed across multiple databases, including Scopus, PubMed, Web of Science, and CINAHL, focusing on studies addressing technological resources aimed at supporting the care and management of Alzheimer's disease.

**Results:**

A total of 23 studies were included in the final analysis. The most frequently utilized technologies were mobile applications and wearable devices. The most identified functionalities included cognitive training, location tracking, task reminders, communication support, fall detection, and vital signs monitoring, often integrated into comprehensive solutions to enhance patient care and safety.

**Conclusion:**

Overall, these technologies were designed to support both patients and caregivers. However, despite the clear benefits and innovative potential of these technologies, significant limitations remain, particularly the lack of empirical validation in real-world clinical settings and the need to ensure greater usability for older adults and individuals with cognitive impairments.

## Introduction

The human brain, which contains over 60 billion nerve cells, is one of the most sophisticated biological systems.^1^ However, diseases such as dementia compromise this complex biological network, disrupting cellular communication and resulting in a rapid and profound deterioration of cognitive functions.^2,3^ According to the World Health Organization, dementia is currently the seventh leading cause of death and one of the main causes of disability and dependency among older adults, affecting more than 55 million people worldwide, with a new case occurring somewhere in the world every 3 seconds.^4^ As a result of population growth and increased life expectancy, it is estimated that by 2030, more than 75 million people will be living in this condition, with this number expected to triple by 2050.^2,4,5^

Dementia is a syndrome characterized by cognitive decline and loss of functional abilities, commonly caused by neurodegenerative diseases, most notably Alzheimer's disease (AD).^2^ Alzheimer's disease is the most prevalent form of dementia, accounting for 60–70% of cases, and is rapidly becoming one of the most costly, lethal, and burdensome diseases of this century. It carries immeasurable physical, psychological, social, and economic impacts not only for those diagnosed but also for their caregivers, families, and society.^6^ Although there is currently no cure for dementia or Alzheimer's disease, it is essential to pursue alternative approaches that focus on slowing disease progression and ensuring quality of life for both people living with the condition and those who care for them.^7,8^

In the healthcare context, research has shown that technology plays a crucial role by introducing new possibilities across various settings.^9–11^ Mobile health applications (apps), websites, wearable devices, and virtual and augmented reality systems are being developed to remotely monitor, guide, and support the daily lives of people with Alzheimer's and their caregivers.^7,8,12,13^ Technology presents numerous potential applications in the context of dementia, ranging from diagnosis and assessment to care delivery and support for ageing in place.^12,13^ Its use has proven effective in stimulating cognitive functions, improving communication, promoting autonomy, and strengthening social bonds for people with dementia.^13–16^ Beyond the direct benefits for people with dementia, technologies have also been recognized as important support tools for caregivers and families.^17,18^ They offer features that facilitate care management and organization, safety monitoring, and even day-to-day tasks such as medication reminders, real-time location tracking, and communication with healthcare professionals.^13,19,20^

In this context, care management becomes a priority, as the progressive course of the disease requires structured and ongoing strategies involving patients, caregivers, and healthcare professionals.^21,22^ The integration of technological resources into care plans has shown great potential to support care coordination, facilitate communication among all involved, and personalize interventions according to the patient's needs.^8,13^

For the purposes of this review, the term ‘technology’ refers specifically to digital and electronic resources, such as mobile applications, wearable devices, web-based platforms, and sensor-based systems, that are designed to support care management.^7,8,13^ Given the expansion of technological resources in this field, the present article aimed to map and describe the technological tools reported in the literature that are intended for care management in Alzheimer's disease, focusing on supporting people living with the condition, their families, caregivers, and healthcare professionals.

Accordingly, this study sought to answer the following research questions:

→ What technological resources have been developed and/or applied in the context of care management in Alzheimer's disease?
→ What are the main functionalities and purposes of these resources in supporting individuals with AD, their caregivers, families, and healthcare professionals*?*

## Methods

### Study design

This study was conducted in accordance with the methodological guidelines of the Joanna Briggs Institute (JBI) for scoping reviews.^23^ The structure of the article followed the PRISMA-ScR (Preferred Reporting Items for Systematic Reviews and Meta-Analyses Extension for Scoping Reviews) checklist,^24^ ensuring transparency in the presentation of the results. The study protocol was registered on the Open Science Framework^®^ platform (DOI 10.17605/OSF.IO/FCS3E).

### Research strategy

The first step was the formulation of the research question using the Population, Concept, and Context (PCC) framework. Once the research question that best described the objectives of this review was defined, a comprehensive literature search was conducted. The final search query was developed after multiple test iterations using a wide range of different combinations. It was constructed using the PCC concepts in English and adapted to the syntax of each of the electronic databases analysed: SCOPUS, PubMed, Web of Science, and CINAHL. The most recent database search was conducted in January 2025. Regarding the *Population*, the terms considered included ‘Alzheimer's patient’, ‘patient with dementia’, ‘Alzheimer's caregiver’, or ‘dementia caregiver’. For the *Concept*, expressions such as ‘technological resource’, ‘mobile application’, ‘wearable technology’, ‘telehealth’, ‘digital health’, ‘assistive technology’, ‘website’, ‘telemedicine’, ‘online application’, ‘mobile health’, ‘digital platform’, ‘mHealth’, and ‘e-health’ were included. Concerning the *Context*, the terms used comprised ‘decision-making’, ‘information’, ‘management’, ‘rehabilitation’, ‘education’, ‘training’, ‘support’, ‘monitoring’, and ‘improvement’.

For example, in PubMed, the search strategy was structured as follows: (‘Alzheimer Disease’[Mesh] OR ‘dementia’[tiab] OR ‘Alzheimer's patient’[tiab] OR ‘dementia caregiver’[tiab]) AND (‘mobile application’[tiab] OR ‘wearable technology’[tiab] OR ‘telehealth’[tiab] OR ‘digital health’[tiab] OR ‘assistive technology’[tiab] OR ‘mHealth’[tiab] OR ‘e-health’[tiab]) AND (‘management’[tiab] OR ‘rehabilitation’[tiab] OR ‘support’[tiab] OR ‘monitoring’[tiab] OR ‘education’[tiab]). Similar strategies were developed for the remaining databases and were adapted as appropriate to the specific syntax and operators of each.

The results obtained from each of these databases were subsequently imported into Rayyan^®^, a web-based platform designed to support the development of systematic and scoping reviews. To ensure a comprehensive and accurate search, each component of the research question included relevant terms and synonyms to broaden the scope of the review.

### Eligibility criteria

Only studies published in English between January 2015 and December 2024 were considered. The eligibility criteria focused on the selection of studies that directly addressed the use of technological resources applied to care management in Alzheimer's disease, according to the PCC framework (Population, Concept and Context). Eligible studies included those targeting people with Alzheimer's disease and/or their formal or informal caregivers in the context of care management. Studies based solely on basic communication technologies, such as video calls, or those not specifically designed for health management, symptom control or rehabilitation in people with Alzheimer's disease were excluded. Short conference abstracts were also excluded.

### Data extraction

The results obtained from the different databases were exported to the Rayyan^®^^25^ platform, where two researchers independently analysed all stages of the selection process. Only the articles that fully met the previously defined eligibility criteria were included in the review. The selection of studies was carried out in different phases. In the first stage, two independent reviewers (MA and CSF) screened the titles and abstracts of all records retrieved in Rayyan^®^. Articles that clearly did not meet the inclusion criteria were excluded at this stage. In the second stage, the same reviewers independently assessed the full texts of potentially eligible studies. Any discrepancies in the selection process were resolved through discussion and consensus with a third researcher (MCF). Data collection was supported by a database specifically designed for this purpose, allowing for a clear and structured organization of the information. This included: study identification details (author, year, country, and title), methodological characteristics (study type and objectives), information on the technology used (type and resource applied), context of application, as well as the number and profile of participants (gender, role, and involvement in the study), along with the main results and reported limitations. To ensure transparency and systematic presentation of the data, the PRISMA-ScR structure was adopted to guide the organization of information throughout the process.

### Data analysis

The data were systematised and presented in a descriptive and narrative manner, using tables and figures to facilitate the reading and understanding of the main findings of the review. The extracted information was organized according to the methodological characteristics of the studies, the types of technologies identified, their functionalities, and the participant profiles, allowing the identification of patterns. The main findings were presented in supporting tables and figures, facilitating an overall understanding of the synthesized evidence.

## Results

**Figure 1.**
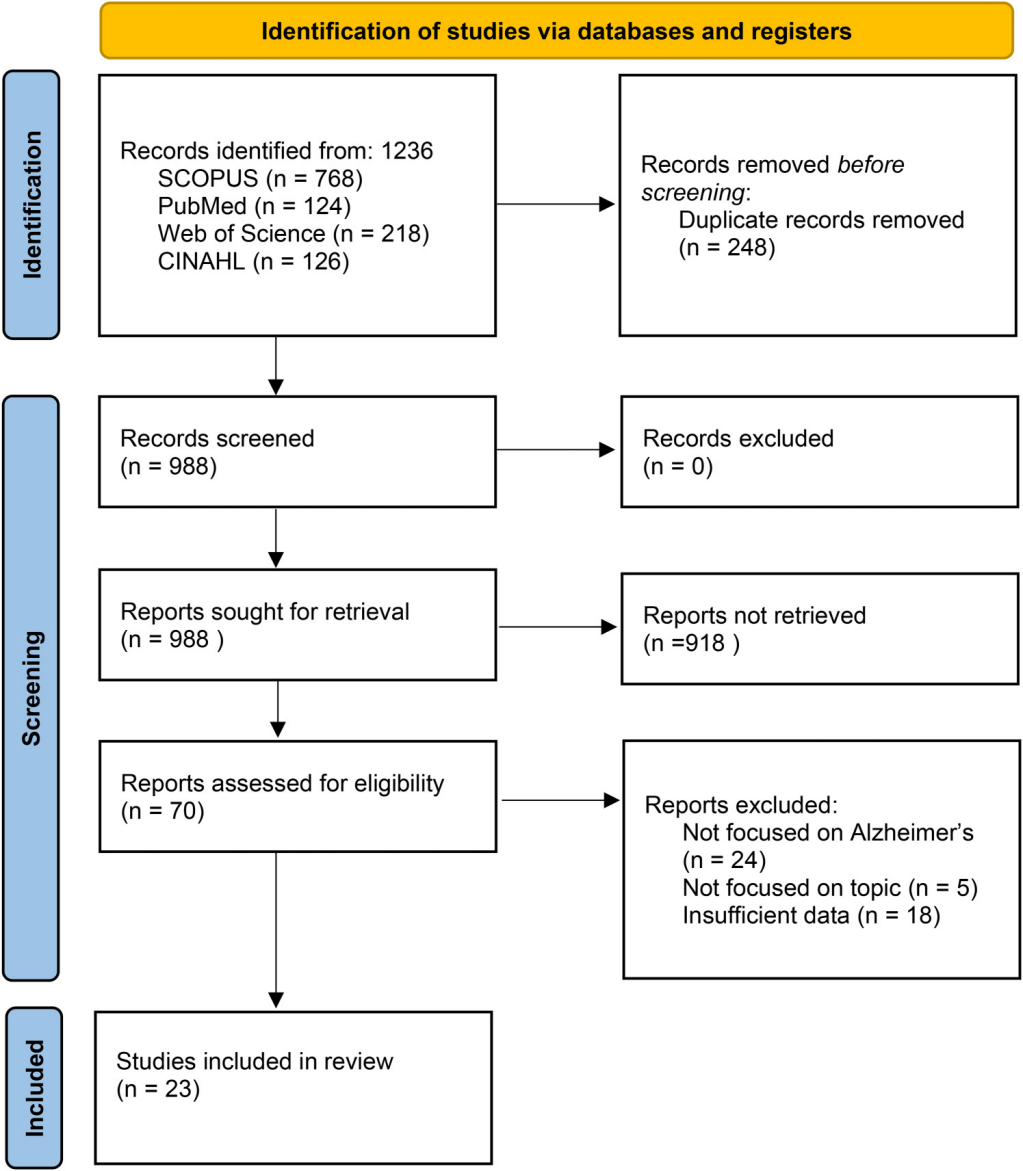
PRISMA – diagram flow (2020).

In total, 1236 records were identified, of which 248 were removed as duplicates, leaving 988 for title and abstract screening. After the exclusion of 918 records and the full-text assessment of 70 articles, 23 studies met the inclusion criteria and were incorporated into the review, as illustrated in Figure 1.^14–16^^,18–20,26–42^

Table 1 presents the main data extracted from the 23 studies included, according to the items previously referenced.

**Table 1.** Characteristics of the included articles

Authors Year Country	Study design	Objective	Test participants					Technology			Study results	Limitation
			N	Age Gender Disease	Patient	Caregiver	Health professional	Types of technology	Description	Features		
Kadhim et al. (2023)^26^ Tunisia	Pilot Study	To develop a real-time face recognition system to assist Alzheimer's patients in identifying people around them, using glasses	None	-	-	-	-	Wearable	Camera in glasses with built-in Wi-Fi	Facial Recognition Communication Support	The prototype achieved 99.46% training accuracy and 99.48% face recognition accuracy	No empirical testing; Dependent on users consistently carrying the wearable; Affected by device compatibility, lighting, and facial occlusions
Mohan et al. (2023)^27^ India	Pilot Study	Mobile App for recording and summarizing Alzheimer patients’ conversations using face recognition and NLP	None	-	-	-	-	Mobile App	Voice capture and conversation summarization	Facial Recognition Communication Support Speech Recognition	The app stores one-on-one conversations, improving Alzheimer's patients’ independence.	No empirical testing; Privacy concerns with conversation storage; Usability issues in the elderly
Weerakoon et al. (2018)^28^ Sri Lanka	Pilot Study	To develop a cognitive training app with personalized exercises targeting motor skills, communication, memory, and written language	NS	NS	**√**	-	-	Mobile App	Mobile cognitive and memory support	Cognitive Training Cognitive Tracking	The system was systematically developed to support Alzheimer's patients in memory improvement	Test with Patients, Healthy controls, and Suspected Alzheimer's cases. User proficiency varies
Alhassan et al. (2017)^29^ Saudi Arabia	Pilot Study	App for activity reminders and assessment tools for Alzheimer's patients	None	-	-	-	-	Mobile APP + Wearable	Wristband sensor and the captured data will be accessed via Application Programming Interface	Cognitive Training Cognitive Tracking Daily Tasks Reminders Health Signals Monitoring	The system offers activity reminders and assessment tools for caregivers.	No empirical testing; Prototype stage; Usability issues in the elderly
Aljojo et al. (2020)^30^ Saudi Arabia	Pilot Study	Mobile app integrating reminders, GPS tracking, and facial recognition	354	*Caregivers* (*N* = 177): 26–40 years 81% female, 19% male *Patients* (*N* = 177) 61 and 90 years 66% female, 34% male	**√**	**√**	-	Mobile App	Mobile app with location tracking, reminders, caregiver support	Location Tracking Daily Tasks Reminders	The app builds patient confidence, fosters social engagement, and supports caregivers through technologies like face recognition	Facial recognition and hardware limitations; Lacks caregiver monitoring features
Vergara et al. (2015)^31^ Colombia	Pilot Study	Mobile health app for geolocation and safety tracking	None	-	-	-	-	Mobile App	Internet of Things-based mobile tracking system for Alzheimer's monitoring	Location Tracking Safety Alerts Point Of Interest Finder	Caregivers can monitor patients remotely via mobile devices.	No empirical testing; High GPS battery consumption
Acharya et al. (2016)^14^ India	Pilot Study	Develop a mobile app to support Alzheimer's patients by enhancing safety, providing memory aids, and assisting caregivers.	NS	NS	-	-	**√**	Mobile App	*Conceptual mobile app guided by medical input*	Cognitive Training Location Tracking Navigation Support Fall Detection Safety Alerts	The app provides essential functionalities to meet users’ needs and aims to raise awareness about digital healthcare	Health Professionals interviews No empirical testing; Re-quires internet and device sensors
Siddiq et al. (2018)^16^ Pakistan	Experimental Study	Mobile app combining cognitive therapy, reminders, and tracking in Urdu	20	*Patients*: 20 (mild to moderate stage) Elderly (not specified in detail) *Caregivers*: Participated, but exact number not specified	**√**	**√**	-	Mobile App	Assistive reminder app for Alzheimer care	Cognitive Training Location Tracking Daily Tasks Reminders Daily Tasks Tracking	CareD integrates cognitive therapies, monitoring, and reminders to reduce stress and improve dementia care.	Limited to Urdu language and Android
Eichhorn et al. (2018)^32^ Germany	Experimental Study	Gamified activities to stimulate memory, sensory engagement, and social interaction	15	10 Alzheimer's patients (All elderly, but exact ages are not reported) 5 caregivers	**√**	**√**	-	Mobile App	*Cognitive support app concept for dementia care*	Cognitive Training Cognitive Tracking	Gamification enhances cognitive abilities and daily functioning in Alzheimer's patients.	Scalability and adaptation challenges
Siangpipop et al. (2023)^33^ Thailand	Pilot Study	User-friendly application for tracking and monitoring Alzheimer's patients	5	NS	-	**√**	-	Mobile App	Mobile app supporting caregivers in daily Alzheimer's care management	Location Tracking Daily Tasks Reminders Safety Alerts	The movement tracking app was developed using design thinking to improve user experience for AD patients.	Small sample size limits design assessment
Duque et al. (2016)^34^ Spain	Case Study	Develop a method to link smartphone movement data with Alzheimer's disease stages.	35	7 Early stage 18 Middle stage 10 late stage	**√**	-	-	Wearable	Mobile phone accelerometer data analyzed to classify Alzheimer's stage using neural networks	Cognitive Tracking Location Tracking Fall Detection	The smartphone-based neural network classified Alzheimer's movement patterns with 83% accuracy.	Lacks integration with other sensors; Accuracy depends on capture device quality
Byeon et al. (2019)^18^ Korea	Descriptive Study	Develop a machine learning model to predict depression in Alzheimer's caregivers via a mobile app.	2592	1154 males 1438 females	-	**√**	-	Mobile App	Random forest-based depression prediction app for caregivers	Depression Prediction	Random Forest revealed key predictors of caregiver depression, highlighting machine learning's role	Survey based data may lack personalization
Zhang et al. (2023)^35^ Korea	Pilot Study	App for memory exercises, reminders and routine management	70	All over 60 years old	**√**	-	-	Mobile App	Mobile app with games, reminders, tracking	Cognitive Training Location Tracking Daily Tasks Reminders	The mobile app with a user-friendly interface improves cognitive abilities in older users, encouraging engagement in cognitive training	The app was evaluated through surveys and interface simulations but not used in daily living contexts by elderly users. Limited applicability
Aljehani et al. (2018)^36^ Taiwan	Pilot Study	Develop an IoT system using Apple Watch to monitor and support Alzheimer's patients	36	NS	-	-	-	Mobile APP + Wearable	Apple Smartwatch connected with IOS app	Location Tracking Daily Tasks Reminders Health Signals Monitoring Educational Tools Safety Alerts	iCare integrates IoT with Apple smartwatches to enhance Alzheimer's patient safety and task management.	Limited to Apple devices; Internet required for data sync
Biswas et al. (2021)^15^ Bangladesh	Pilot Study	Design an indoor navigation system using BLE beacons and Wi-Fi for Alzheimer's patients.	None	-	-	-	-	Mobile APP	Combination of wall-mounted wireless sensors, a mobile app and WiFi/Bluetooth beacons	Location Tracking Navigation Support	Real-time remote monitoring system supports families and clinicians.	No testing; inaccurate RSS, signal interference, security risks, and user interaction required.
Rashmi et al. (2023)^37^ India	Pilot Study	Wearable device to monitor Alzheimer's patients’ vitals, fall detection, and location tracking	13	13 healthy volunteers Aged 22-33	-	-	**√**	Wearable	Wearable device with biometrics, fall detection, GPS tracking	Location Tracking Fall Detection Health Signals Monitoring	The wearable device monitors Alzheimer's patients’ biometrics and GPS location, achieving 93% fall detection sensitivity and 95% specificity	No empirical testing; Dependent on Wi-Fi; Requires sensor calibration; Limited battery life
Duarte et al. (2023)^38^ Portugal	Pilot Study	Mobile app to monitor and manage dietary plans for Alzheimer's patients, providing real-time feedback to nutritionists	20	60–70 years (mean 64.8) 14 female, 6 male	-	**√**	-	Mobile App	Mobile app for food tracking and caregiver support	Daily Tasks Tracking	The mobile app simplifies meal tracking and nutritional monitoring.	Does not address mobility or communication aspects ofcare
Amaro et al. (2024)^39^ Italy	Pilot Study	Dynamic and personalized serious game to enhance spatial and autobiographical memory in Alzheimer's patients.	None	-	-	-	-	Mobile APP + Website	Personalized serious game for memory rehabilitation	Cognitive Training Cognitive Tracking	MoM is a cognitive rehabilitation tool designed to slow memory decline and improve emotional well-being.	No empirical testing Intended for Alzheimer's patients (early to moderate stage)
Francis et al. (2024)^40^ USA	Pilot Study	AI-driven mobile app supporting Alzheimer's caregivers by identifying patient personality and guiding daily care interactions	16	Females Aged 20-48	-	**√**	-	Mobile App	Affective AI mobile app simulating identity-based social interactions.	Communication Support	VIPCare uses AI to support emotionally intelligent dementia care, aiming to improve well-being and reduce caregiver stress.	Residents were not directly assessed due to cognitive impairment; profiles relied on caregiver reports and were impacted by COVID-19.
Fardoun et al. (2017)^20^ Saudi Arabia	Pilot Study	A mobile-cloud-based architecture using face recognition for Alzheimer's patients to identify and recall familiar people	41	23 males 18 females Aged 55-72	**√**	-	-	Mobile APP + Wearable	Real-world scenario with smartwatch and mobile app for face recognition	Facial Recognition Communication Support	The cloud-based system helps Alzheimer's patients recognize familiar people, but usability issues remain.	Usability issues in the elderly; Dependent on network and hardware limitations
Chaudhry et al. (2021)^41^ USA	Pilot Study	Mobile app designed to help dementia patients and caregivers manage daily tasks, cognitive stimulation, and communication	5	-	-	**√**	-	Mobile App	Tablet app for memory and caregiver interaction support	Cognitive Training Daily Tasks Reminders	RefineMind supports dementia care with memory aids and communication tools.	Usability issues in the elderly; Requires internet; Limited clinical validation
Lobo et al. (2023)^42^ India	Pilot Study	Wearable IoT device providing GPS tracking, health monitoring, and reminders for Alzheimer's patients	None	-	-	-	-	Mobile APP + Wearable	Real-time location tracking (via GPS), health monitoring (heartbeat, BP), food/medication reminders	Location Tracking Daily Tasks Reminders Health Signals Monitoring Safety Alerts	Enhances patient safety with location tracking, health monitoring, and reminders.	No empirical testing; Location updates every 30 min; Dependent on internet for real time tracking
Jimenez et al. (2024)^19^ Peru	Experimental Study	Energy-efficient wearable shoe device for geolocation using piezoelectric energy harvesting	1	NS	-	-	**√**-	Mobile APP + Wearable	Shoe developed with a LoRa technology	Location Tracking Safety Alerts	The geolocation system successfully tracks Alzheimer's patients in real time.	Tested by one user; not with Alzheimer's patients. Limited range and battery issues

APP + Wearable – mobile application integrated with a wearable device; APP + Website – mobile application integrated with a web platform; Mobile APP – mobile application; BP – blood pressure; GPS – Global Positioning System; IoT – Internet of Things; LoRa – long range (low−power, wide-area wireless communication protocol); BLE – Bluetooth Low Energy; RSS – received signal strength; AD – Alzheimer's disease; iCare – integrated care application; NLP – Natural Language Processing; AI – artificial intelligence; MoM – Mosaic of Memory (serious game); CareD – Cognitive Assistance and Reminder Device; IOS – Apple Operating System; *N* – number of participants; NS – not specified; √ – present/included

### Characteristics of the included studies

A total of 23 articles published between 2015 and 2024 were included. Most studies were published in 2023.^26,27,33,35,37,38,42^ Although a sharper increase in publications might have been expected following the 2020 pandemic, the data indicate that interest in technology-based solutions for Alzheimer's disease had already been growing, reflecting the advancement of digital innovation.^43,44^ In terms of methodological design, most studies were exploratory or descriptive in nature, with a predominance of pilot studies, commonly used to validate prototypes or assess usability during early stages of technological development.

### Characteristics of participants

Regarding participants, the 23 included studies involved a total of 3405 individuals. The number of participants per study varied widely, ranging from 1^19^ to 2592.^18^ However, seven studies did not conduct user testing (*n* = 7),^15,26,27,29,31,39,42^ limiting their findings to prototype descriptions without validation by the target population. One additional study^14^ did not specify whether user testing was performed (NS).

Participant profiles also varied considerably, reflecting the diverse objectives of the technological solutions analyzed. Three main recipient groups were identified: patients (*n* = 7),^16,20,28,30,32,34,35^ caregivers (*n* = 8),^16,18,30,32,33,38,40,41^ most of whom were family members, and healthcare professionals (*n* = 3).^14,19,37^

Regarding demographic information, not all studies have reported complete data on age, gender or participant profiles, which limits the possibility of systematic comparisons. Where such information was available, most patients were older adults aged over 60 years, with some studies reporting mean ages between 64 and 70 years. Several studies also highlighted a predominance of female participants, both among patients and caregivers.

### Characteristics of technological resources

**Figure 2.**
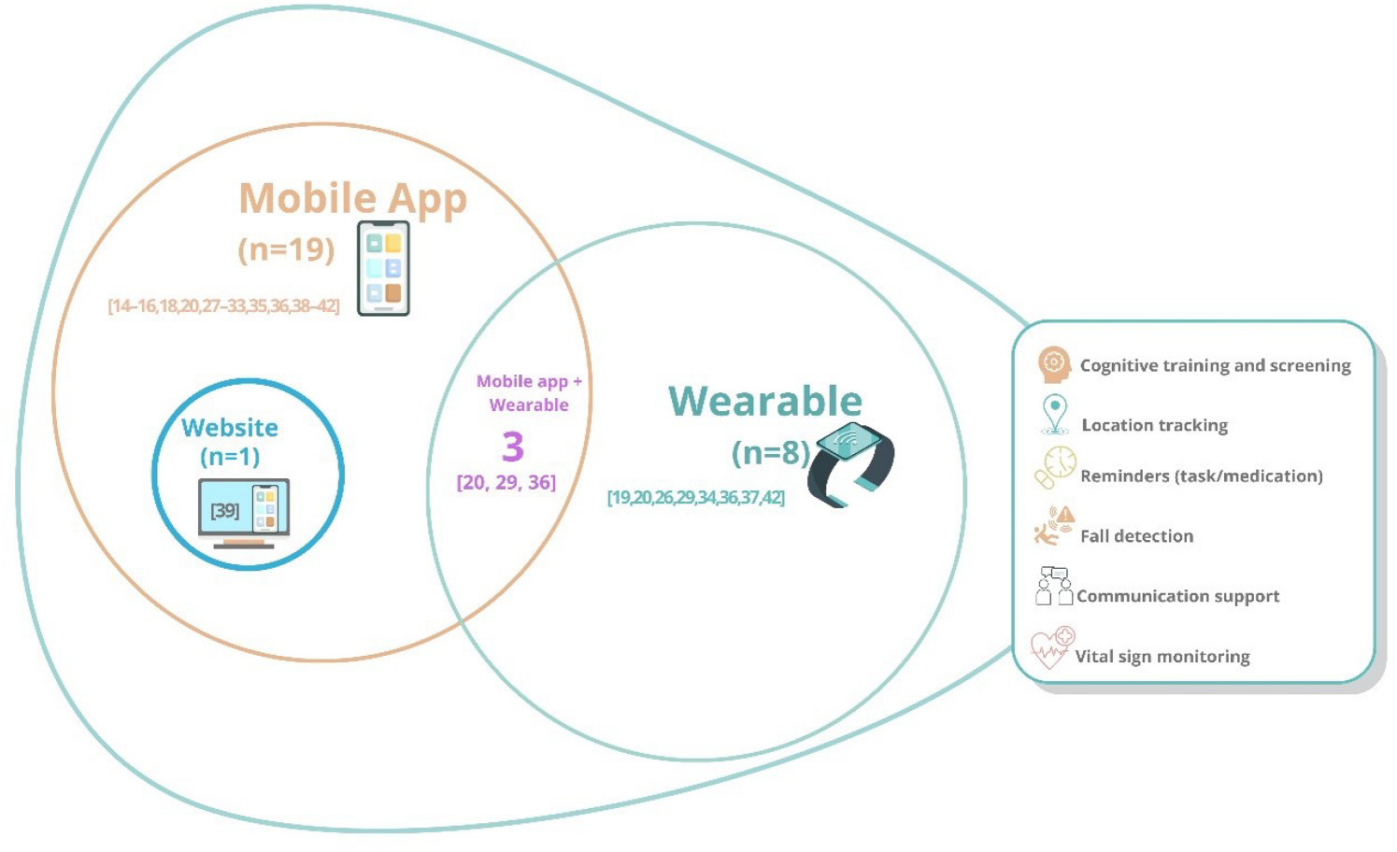
Distribution of studies according to the type of technology used.

The analysis of the studies included revealed a wide range of technological solutions. The most used technology was the mobile application, reported in most studies (*n* = 20).^14–20^^,27–33,35,36,38–42^ (Figure 2)

These applications were developed for various purposes, including cognitive training, daily task reminders, real-time location tracking, caregiver communication support, and health monitoring. In addition, integration with wearable devices was identified in eight studies,^19,20,26,29,34,36,37,42^ involving smartwatches, wristbands, sensorized vests, and sensor-equipped shoes. Although less frequent, one study included a complementary web-based platform integrated with a mobile app,^39^ consisting of a personalized serious game for memory training. This illustrates the potential of hybrid approaches that combine mobile and online digital technologies for therapeutic purposes.

The most frequent functionalities found across the technological solutions included cognitive training and screening,^14,16,18^^27–30^^,32,35,38–41^ GPS or Bluetooth beacon-based location tracking,^14–16^^,19,20,28,30,31,34–37,42^ task and medication reminders,^16^^29–31^^,33,35,36,41,42^ fall detection,^14,34,37^ communication support,^20,26,27,40^ and vital sign monitoring.^29,36,37,42^

## Discussion

This scoping review enabled the mapping and characterization of technological resources developed within the context of care management in Alzheimer's Disease (AD). The technological tools identified were targeted toward three main user profiles: patients diagnosed with Alzheimer's disease,^19,20,28,30,31^^34–39^ informal or family caregivers,^18,30,32,33,38,40,41^ and, to a lesser extent, healthcare professionals.^14,19,37^

Patients were involved in several studies, either as direct end-users or as prototype testers, contributing to the evaluation of usability and potential clinical benefits.^20,28,30,31,34,35^ The inclusion of the lived experience of people with the disease provides unique insights into how digital technologies should be developed.^28,30^ Regarding caregivers, their involvement was often limited to usability testing rather than co-design,^18,30,32,33,38^ despite their perspectives being crucial to ensuring adherence, acceptability, and the integration of these tools into the routines of patients and families. Healthcare professionals were included in only three studies,^14,19,37^ and even then, their participation was mainly restricted to conceptual design or validation stages. This limited involvement highlights a significant gap, since professionals are also key actors in clinical implementation and can bridge the divide between technological innovation and clinical practice. Overall, these findings suggest that future research should aim for a more balanced and active involvement of all three groups.

Regarding the temporal distribution of publications, most studies were published in 2023,^26,27,33,35,37,38,42^ reflecting a growing interest from the scientific community in this field. Nevertheless, the growth of evidence has been gradual and irregular over the past decade, with no continuous progression of publications since 2015.

The expectation of a sharper increase in publications after 2020 is consistent with the accelerated adoption of digital technologies during the COVID-19 pandemic.^43,44^ However, despite this global trend, the number of studies addressing the management of Alzheimer's disease through digital solutions has remained limited. This suggests that although innovations in digital health have expanded rapidly, their application in this context may have encountered additional barriers, including ethical considerations, usability challenges, and the complexity of adapting technologies to populations with cognitive impairment.

The 23 included studies revealed a growing diversity of digital solutions, with an emphasis on mobile applications, reflecting the advancement of technological innovation and findings consistent with other studies in different populations.^11,45,46^ Among the technological resources identified, mobile applications were the most frequently reported technologies.^14–16^^,18–20,27–33,35,36,38–42^ These applications varied in terms of structure and functionality and were designed for mobile devices such as smartphones and tablets. Some applications were standalone tools, while others were integrated with external sensors or cloud-based platforms.^20,27,29,36,42^

Integration with wearable devices was reported in eight studies,^19,20,26,29,34,36,37,42^ taking various forms such as smartwatches,^20,36^ wristbands,^29^ sensorized vests,^37^ and GPS-enabled footwear.^19^ Communication between wearable devices and mobile applications occurred, in most cases, through Bluetooth or Wi-Fi connectivity,^20,26,29,42^ enabling real-time transmission of data such as location, movement patterns, vital signs, and automated alerts.^19,36,37,42^

Wearable devices represent a promising strategy for managing various conditions, although their acceptance still depends on overcoming barriers related to comfort, usability, and emotional impact on patients.^47,48^

This scoping review identified a range of functionalities integrated into technological resources, often combined to optimize support for both the patient and the family caregiver. Cognitive training and screening were one of the functionalities identified,^14,18,27,28,30,35^^38–40^ particularly in mobile applications offering personalized, gamified, and adaptive exercises aimed at stimulating memory, attention, language, and other cognitive functions. These tools were used for both cognitive rehabilitation and early screening of cognitive decline.

Location tracking was another widely reported functionality,^14–16^^,19,20,28,30,31,34–36,42^ primarily implemented through GPS and Bluetooth beacons, enabling real-time geolocation and the use of geofencing to alert caregivers when patients move beyond predefined safe zones. Devices such as smartwatches, sensor-embedded shoes, and integrated mobile systems enhanced safety and continuous monitoring.

Task and medication reminders were also a recurring feature in the analyzed articles,^16^^29–31^^,35,36^ aiming to support treatment adherence and the organization of daily routines.

Fall detection^14,34,37,42^ was implemented using inertial sensors embedded in wearable devices, capable of identifying abrupt movement patterns and automatically issuing alerts.

Regarding communication support,^27,30,40^ this functionality involved technologies such as facial recognition, voice command systems, and artificial intelligence. These solutions aimed to facilitate social interaction and assist in the recognition of family members and caregivers.^30,40^

The functionality of vital signs monitoring^29,36,37,42^ was predominantly associated with the use of wearable devices such as smartwatches and wristbands, allowing for continuous tracking of physiological parameters such as heart rate, sleep quality, and blood pressure. The availability of these real-time data provides critical information for clinical follow-up and supports decision-making by caregivers and healthcare professionals.^29,36^

The studies reviewed highlight a growing trend in the adoption of integrated digital solutions, with a strong emphasis on mobile applications and wearable devices^19,20,26,29,34,36,37,42^ that incorporate multiple functionalities aimed at optimizing the care of patients with Alzheimer's disease and offering comprehensive support to caregivers.

Although the reviewed articles provide valuable insights into technological solutions for Alzheimer's disease care, several aspects highlight the existing challenges and gaps in this field. An important concern is that many of the identified tools did not specify whether their design considered suitability for people with specific needs, low digital literacy, or older adults.^14,26,29,31,39,42^ Aspects such as simplified interfaces, the use of larger fonts, or cultural adaptation of content were not described, which may limit the inclusiveness and applicability of these technological solutions in real-world settings.^14,26,29,42^

Some solutions also rely heavily on continuous device usage,^19,20,36,37,42^ which may compromise their effectiveness, particularly given the known barriers to technology adherence among older adults. Technical challenges are also frequently reported. The accuracy of facial recognition, voice commands, and location tracking can be adversely affected by factors such as lighting conditions, facial occlusion, sensor quality, and signal interference from Wi-Fi, BLE, or LoRa networks.^15,19,20,30,31,34,36,40^ Certain systems exhibit high battery consumption and require constant internet connectivity, limiting their usability in specific environments.^19,29,36,42^ For example, navigation systems often do not account for the dynamic nature of real-world settings, variability in GPS accuracy, or challenges posed by dense urban areas and locations with limited connectivity. Ethical and privacy issues are also of concern, particularly when sensitive data such as voice recordings or location history are stored and processed,^15,19,20,30,31,34,36,40^ as highlighted in recent literature addressing surveillance, consent, and data governance.^49,50^

## Limitations

This study presents several limitations that should be acknowledged when interpreting its findings. Most studies remain at the prototype stage, were conducted with small and homogeneous samples, or were not tested in real patients or clinical environments. These factors limit the empirical validation of their applicability and effectiveness. The lack of robust clinical trials and real-world validation underscores the need for future research to ensure the feasibility and effectiveness of these technologies in supporting patients living with Alzheimer's disease. In addition, this scoping review is subject to methodological limitations, including the potential exclusion of relevant sources due to the restriction to studies published in English, as well as the temporal cut-off from 2015 onwards, which may have affected the comprehensiveness and depth of the findings presented.

## Conclusion

This scoping review enabled the mapping and characterization of technological resources developed for care management in Alzheimer's disease, highlighting an expanding landscape of digital solutions aimed at supporting patients diagnosed with the condition, their caregivers, and healthcare professionals. Emphasis was noted on the use of mobile applications and wearable devices. Although these innovative developments are promising, several limitations were identified. Many existing solutions lack sufficient clinical validation. Technical barriers, ethical and privacy implications, and notably, the difficulty older adults, particularly those with cognitive impairment in adopting and trusting smartphones and other technological systems, remain significant challenges in this field.

Therefore, further research is required to overcome technical limitations, validate user effectiveness across diverse populations, and address ethical considerations. The insights gathered in this review not only underscore the potential of digital interventions but also illuminate critical gaps that future research must address to fully realize their benefits in both clinical and everyday care settings.
